# Effect of teacher’s working conditions on voice disorder in Korea: a nationwide survey

**DOI:** 10.1186/s40557-018-0254-8

**Published:** 2018-07-03

**Authors:** Yi-Ryoung Lee, Hyoung-Ryoul Kim, Seyoung Lee

**Affiliations:** Department of Occupational and Environmental Medicine, Seoul St. Mary’s Hospital, College of Medicine, The Catholic University of Korea, 222 Banpo-daero, Seocho-gu, Seoul, 137701 Republic of Korea

**Keywords:** Teacher, Working condition, Sick leave, Working hour, Sleep hour, Voice disorder, Hoarseness

## Abstract

**Background:**

Korean teacher’s working conditions are deteriorating. There is concern about the deterioration of teachers’ health and voice disorder is one of the most common problems. Teacher’s vocal health is important for them and their students. The aim of the present study was to investigate working conditions that may affect voice disorders.

**Methods:**

In all, 79 primary and secondary schools were randomly selected for a nationwide school system survey (*N* = 3345). In 64 schools, 1617 (48.3%) teachers participated via a postal self-report questionnaire from June 2016 to August 2016. After applying inclusion and exclusion criteria, data from 1301 teachers’ were used for analysis. Multiple logistic regression was used to investigate the associations between general, work-related factors, and frequent voice disorders (fVDs) to estimate the adjusted odds ratio(aOR).

**Results:**

Teachers who reported voice symptoms more than once a week (fVD) made up 11.6%. In a multiple logistic regression, fVD was significantly associated with female, difficulty in applying for sick leave as needed, music teachers (primary school), and less than 6 h of sleep per day (primary school). The aOR for fVD was 2.72 (0.83–8.10) in the longest working hours group (> 52 h/wk) among the primary school teachers, and 1.90 (0.80–4.73) in the longest class hour group (≥ 20 h/wk), 1.52 (0.90–2.62) in homeroom teachers among the secondary school teachers, but not statistically significant.

**Conclusions:**

Korean teachers’ working conditions are associated with fVDs. The school health system must take steps to prevent and treat voice disorders of teachers.

## Background

Recently, it has been reported that Korean teacher’s working hours and workload are increasing. This is explained by the increase in administrative tasks, after-school classes and out-of-class activity, in addition to basic academic duties [1]. There is growing concern over the deterioration of teachers’ health. The International Labour Organization (ILO) and several studies from others have reported that teachers are at risk for conditions such as infection, respiratory and musculoskeletal disorders, poor mental health, burn out, miscarriage, violence and voice disorders [2–6].

Voice disorder (VD) has been one of the most common problems among teachers. Many studies have reported that teachers have more frequent and higher risk of VDs. A U.S. study comparing teachers with non-teachers for life-time prevalence of VDs found that the adjusted odds ratio was 2.04 (1.55–2.68) [7]. The Korean National Health Insurance Service(NHIS) reported in 2014 that the number of teachers who received vocal nodule treatments was about four times higher than that of the total population (460 per 100,000 people and 195 per 100,000 people, respectively) [8]. A review of VDs in 2014 concluded that teachers experienced such a disorder two-to-three times more frequently than the general population [9].

Teachers’ vocal health is important for them and their students. VDs can negatively affect teacher’s work ability [10, 11], quality of life [12, 13] and job loss [11]. Teachers with VDs are less effective with regard to student’ achievement and in developing relationships with their students [14, 15]. Some studies report that the proportion of voice-related absenteeism in teachers was 12–27%, which is significantly higher than for non-teachers [10, 16–18]. Furthermore, the estimated cost in terms of lost workdays and treatment of VDs of U.S. teachers amounted to about $2.5 billion annually [19].

While many studies have been published on teachers’ VDs, few have investigated the association between VD and working conditions such as class hours, working hours, homeroom teacher duties, and sick leave, especially in Korea. This is the first national study of Korean teachers’ VDs. The purpose of this study is to identify working conditions that may affect VDs and to find points for intervention through nationwide surveys.

## Methods

### Study populations and questionnaire survey

The target population of this study was primary and secondary school teachers in Korea. We identified all schools in Korea through the data from Chamgyoyook Research Institute (CGRI) based on the *Statistical Yearbook of Education 2015* by the Korea Ministry of Education. There were 6253 primary schools, 3239 middle schools, and 2348 high schools. We excluded special-purpose and autonomous high schools. We divided the schools into eight groups according to type (primary/middle/general high/vocational high) and region (urban/rural). ‘Urban school’ was defined as a school in metropolitan city and ‘rural school’ was defined as a school in county. The eight groups were urban primary schools, urban middle schools, urban general high schools, urban vocational high schools, rural primary schools, rural middle schools, rural general high schools, and rural vocational high schools. We randomly selected eight schools from each group. Because primary schools have six grades and more teachers, 16 schools were randomly selected for each primary school group, for a total of 80 schools. One selected rural vocational high school was excluded from the final group because it operated in conjunction with a general high school. Finally, all teachers from a total of 79 schools were selected for the study population (*N* = 3345).

This study was approved by the Institutional Review Board (IRB) of Seoul St. Mary’s Hospital (IRB registration number: 2016–0433-0001). The survey was conducted from June 2016 to August 2016. In each school, one responsible teacher who was working at each school was appointed by the CGRI. Paper questionnaires were mailed to each school. We asked all the teachers at each school to complete the questionnaire survey and return it to the designated responsible teacher. Responsible teachers gathered the sealed-questionnaires and mailed them to Seoul St. Mary’s Hospital. A total of 1617 (48.3%) teachers in 64 schools out of 3345 teachers in 79 schools participated in the survey.

**Fig. 1:**
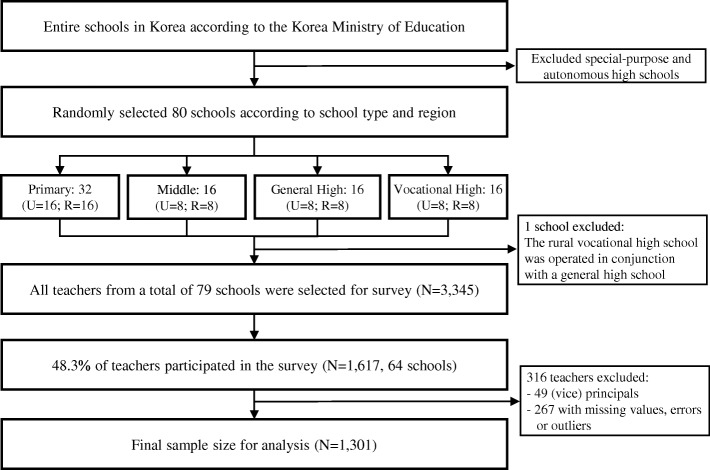
A flow chart depicting study population

The data of 1617 teachers were screened. Principals or vice-principal teachers were excluded (*N* = 49). After screening the data, 267 teachers were excluded due to errors, missing values, or outliers such as over 24 of sleep hours per day, over 100 working hours per week and over 100 students per class. Therefore, 1301 teachers were included in the final analysis (Fig. 1).

### Definition of frequent voice disorder

The self-report questionnaire was designed to investigate sociodemographic characteristics, working conditions, and voice symptoms. Sociodemographic characteristics included age, sex, smoking history, education, sleep hours, and teaching career. Working conditions included the average number of students per class in the current school (number of students), the average class hours per week during this semester (class hours per week), the average working hours per week during this semester (working hours per week), whether they were homeroom teachers, subject taught, whether it was possible to apply for sick leave as needed (sick leave as needed), and employment status. Participants were asked following questions about voice symptoms, “During the last 1 year, have you ever had hoarseness or lost your voice when you did not have a cold?” and to check one of five frequencies: less than once a semester, once a semester, once a month, 2–3 times a month, more than once a week. Frequent voice disorder (fVD) was identified when a teacher responded “more than once a week.”

### Statistical analysis

The number of students and class hours per week were divided considering the *Statistical Yearbook of Education 2016* by the Korea Ministry of Education [20] and average, standard deviation of data from this study. In multiple logistic regression, the reason for differentiating class hours per week between primary and secondary schools was that the percentage of primary school teachers who reported less than 16 h was 5.7%, and that of secondary school teachers who reported 24 h or more was 0.5%. The reason for dividing working hours per week by 40 h and 52 h was that the legal working hours in Korea are fewer than 40, and agreement of the parties allows up to 12 h of extended work (total of 52 h).

We used the t-test for the mean comparison, Pearson’s correlation to yield correlation coefficient, chi-square test for frequency comparison, and multiple logistic regression to obtain the adjusted odds ratio (aOR). Because primary and secondary school teachers differed in general characteristics and working conditions, we stratified them into two groups. The variables included in the multiple logistic regression model were as follows: variables with *P*-value < 0.15 for univariate analysis (sex, smoking, school type, class hours per week, working hours per week, whether they were homeroom teachers, subject taught, and sick leave as needed), and variables which are associated with VD in the previous study (age, sleep hours, and working environment). To adjust the working environment, number of students (to consider the acoustic and noise condition) and region of school were included in the multiple logistic regression model. Because there was a strong correlation between age and teaching career (*r* = 0.93), age was included and teaching career was excluded when performing a multiple logistic regression. The school type was included in the adjustment for secondary school teachers’ data. Smoking was excluded from the adjustment in the analysis of female teachers, and subject taught was additionally excluded in the analysis of female secondary school teachers because the number was too small to be suitable for multiple logistic regression. The current smoker number was 1 in female primary school teachers (0 with fVD) and 0 in female secondary school teachers. Among female secondary school teachers, the numbers of physical education teachers and music teachers with fVD were 0 and 2, respectively.

Two-tailed *p*-values < 0.05 were considered statistically significant. Statistical analysis was performed using SAS software (version 9.4).

## Results

### General characteristics and working conditions

Among the 1301 participants, 558 (42.9%) were primary school teachers and 743 (57. 1%) were secondary school teachers. Compared with primary school teachers, secondary school teachers were older (the average age: 40.28 ± 9.31, 45.66 ± 9.75, respectively, *P* < 0.001), had a higher proportion of current smokers (5.2, 13.7%, respectively. *P* < 0.001), and had more men (20.3, 53.7%, respectively, *P* < 0.001; see Table 1). Class hours per week were longer for primary school teachers (21.23 ± 3.83, 17.13 ± 2.70, respectively, *P* < .0001), but secondary school teachers had longer working hours per week (43.07 ± 5.79, 46.86 ± 8.67, respectively, *P* < 0.001), and a higher proportion of over 52 working hours per week (3.9, 13.5%, respectively, *P* < 0.001; see Table 2). About half of the total teachers had difficulty in applying for sick-leave as needed.

**Table 1:** General characteristics and frequent voice disorder (fVD) of teachers

Variables	Primary school			Secondary school		
	N(%)	fVD		N(%)	fVD	
		N (%)	*P*-value*		N (%)	*P*-value*
Total	558 (100)	71 (12.7)	–	743 (100)	80 (10.8)	
School type						
Primary	558 (100)	71 (12.7)	–	–	–	
Middle	–	–		196 (26.4)	30 (15.3)	0.031^†^
General high	–	–		317 (42.7)	33 (10.4)	
Vocational high	–	–		230 (30.9)	17 (7.4)	
Region						
Urban	326 (58.4)	46 (14.1)	0.244	460 (61.9)	50 (10.9)	0.909
Rural	232 (41.6)	25 (10.8)		283 (38.1)	30 (10.6)	
Teaching career (yrs)						
< 5	85 (15.2)	14 (16.5)	0.343	87 (11.7)	12 (13.8)	0.504
5–14	166 (29.8)	24 (14.5)		174 (23.4)	22 (12.6)	
15–24	190 (34.0)	18 (9.5)		166 (22.4)	17 (10.2)	
≥ 25	117 (21.0)	15 (12.8)		316 (42.5)	29 (9.2)	
Age (yrs)						
< 30	85 (15.2)	14 (16.5)	0.395	47 (6.3)	5 (10.6)	0.368
30–39	176 (31.5)	25 (14.2)		175 (23.5)	25 (14.3)	
40–49	195 (35.0)	19 (9.7)		184 (24.8)	19 (10.3)	
≥ 50	102 (18.3)	13 (12.8)		337 (45.4)	31 (9.2)	
Sex						
Male	113 (20.3)	6 (5.3)	0.008^†^	399 (53.7)	19 (4.8)	<.001^†^
Female	445 (79.7)	65 (14.6)		344 (46.3)	61 (17.7)	
Smoking status						
Never smoker	501 (89.8)	67 (13.4)	0.394	479 (64.5)	68 (14.2)	<.001^†^
Ex-smoker	28 (5.0)	2 (7.2)		162 (21.8)	6 (3.7)	
Current smoker	29 (5.2)	2 (6.9)		102 (13.7)	6 (5.9)	
Sleep hours (hrs/day)						
< 6	67 (12.0)	13 (19.4)	0.058	155 (20.9)	21 (13.6)	0.393
6–7	212 (38.0)	31 (14.6)		359 (48.3)	38 (10.6)	
≥ 7	279 (50.0)	27 (9.7)		229 (30.8)	21 (9.2)	

*by Chi-square test, ^†^Statistically significant (*P* - value < 0.05)

**Table 2:** Working conditions and frequent voice disorder (fVD) of teachers

Variables	Primary school			Secondary school		
	N(%)	fVD		N(%)	fVD	
		N (%)	*P*-value*		N (%)	*P*-value*
Total	558 (100)	71 (12.7)	–	743 (100)	80 (10.8)	–
Number of students (N/class)						
< 22	115 (20.6)	11 (9.6)	0.651	57 (7.7)	8 (14.0)	0.249
22–24	225 (40.3)	32 (14.2)		42 (5.7)	5 (11.9)	
25–27	207 (43.4)	27 (13.0)		270 (36.3)	21 (7.8)	
≥ 28	11 (2.0)	1 (9.1)		374 (50.3)	46 (12.3)	
Class hours (hrs/wk)						
< 16	32 (5.7)	2 (6.3)	0.566	153 (20.6)	10 (6.5)	0.060
16–19	33 (5.9)	5 (15.2)		457 (61.5)	49 (10.7)	
20–23	415 (74.4)	56 (13.5)		129 (17.4)	21 (16.3)	
≥ 24	78 (14.0)	8 (10.3)		4 (0.5)	0 (0)	
Working hours (hrs/wk)						
40	213 (38.2)	20 (9.4)	0.034^†^	125 (16.8)	13 (10.4)	0.988
41–52	323 (57.9)	45 (13.9)		518 (69.7)	56 (10.8)	
> 52	22 (3.9)	6 (27.3)		100 (13.5)	11 (11.0)	
Homeroom teacher						
Yes	460 (82.4)	57 (12.4)	0.609	404 (54.4)	53 (13.1)	0.024^†^
No	98 (17.6)	14 (14.3)		339 (45.6)	27 (8.0)	
Subject						
Physical education	18 (3.3)	1 (5.6)	0.003^†^	29 (3.9)	2 (6.9)	0.789
Music	11 (2.0)	5 (45.5)		19 (2.6)	2 (10.5)	
Others	521 (94.7)	65 (12.3)		690 (93.5)	76 (11.0)	
Sick leave as needed						
Yes	295 (52.9)	27 (9.2)	0.007^†^	417 (56.1)	26 (6.2)	<.001^†^
No	263 (47.1)	44 (16.7)		326 (43.9)	54 (16.6)	
Employment status						
Regular	540 (96.8)	67 (12.4)	0.219	638 (85.9)	70 (11.0)	0.657
Part-time	18 (3.2)	4 (22.2)		105 (14.1)	10 (9.5)	

*by Chi-square test, ^†^Statistically significant (*P* - value < 0.05)

### Frequency of frequent voice disorder

In both primary and secondary school teachers, teachers who had difficulty in applying for sick leave as needed and women were more likely to experience fVD. In primary school teachers, the frequency of fVD increased as working hours increased (*P* = 0.034) and was higher in the group working the longest hours (> 52 h/wk.; 27.3%). The frequency of fVD of middle school teachers was significantly higher than that of general and vocational high school teachers (Table 1). In secondary school teachers, the frequency of fVD was significantly higher in homeroom teachers than non-homeroom teachers (13.1, 8.0%, respectively, *P* = 0.024) and tended to increase with increasing class hours.

### Results of multiple logistic regression

Table 3 shows the results of multiple logistic regression for primary school teachers. The aOR (95% CI) for fVD was 1.71 (1.00–2.98) for teachers who had difficulty in applying for sick leave as needed and was 6.76 (1.52–30.75) for music teachers. Sleep hours were significant after adjustment: < 6 h/day, aOR = 2.24 (1.02–4.73). As working hours increased, aOR tended to increase but was not statistically significant. Analysis of only female primary school teachers showed similar results.

**Table 3:** Results of multiple logistic regression for frequent voice disorder (fVD): primary school teachers

General and work-related factors	Total		Female	
	aOR^a^	95% CI	aOR^b^	95% CI
Age (yrs)				
< 30	1		1	
30–39	0.98	(0.46–2.12)	0.84	(0.39–1.88)
40–49	0.56	(0.26–1.26)	0.53	(0.24–1.20)
≥ 50	0.79	(0.33–1.90)	0.66	(0.27–1.63)
Sex				
Male	1		–	
Female	2.78	(1.01–9.66)^c^	–	
Smoking status				
Nerver smoker	1		–	
Ex-smoker	1.14	(0.16–5.05)	–	
Current smoker	1.31	(0.17–6.99)	–	
Sleep hours (hrs/day)				
< 6	2.24	(1.03–4.77)^c^	2.34	(1.03–5.14)^c^
6–7	1.69	(0.93–3.05)	1.69	(0.91–3.13)
≥ 7	1		1	
Class hours (hrs/wk)				
< 20	1		1	
20–23	1.40	(0.53–4.17)	1.61	(0.57–5.25)
≥ 24	1.26	(0.35–4.56)	1.40	(0.36–5.67)
Working hours (hrs/wk)				
40	1		1	
41–52	1.25	(0.69–2.33)	1.21	(0.65–2.34)
> 52	2.72	(0.83–8.10)	2.42	(0.67–7.87)
Homeroom teacher				
Yes	0.94	(0.40–2.45)	0.80	(0.33–2.12)
No	1		1	
Subject				
Physical education	0.72	(0.04–4.95)	1.27	(0.06–10.31)
Music	6.76	(1.52–30.75)^c^	4.66	(0.93–22.94)
Others	1		1	
Sick leave as needed				
Yes	1		1	
No	1.71	(1.00–2.98)^c^	1.76	(1.00–3.16)^c^

*aOR* adjusted odds ratio, *95% CI* 95% confidence interval

^a^Adjusted for age, sex, smoking, region, number of students, sleep hours, class hours, working hours, homeroom teacher, subject, and sick leave as needed

^b^Adjusted for age, region, number of students, sleep hours, class hours, working hours, homeroom teacher, subject, and sick leave as needed

^c^Statistically significant (*P* < 0.05)

Table 4 shows the results of multiple logistic regression for secondary school teachers. The aOR (95% CI) was 2.40 (1.41–4.16) for teachers who had difficulty in applying for sick leave as needed and was 1.53 (0.91–2.64) for homeroom teachers. For female homeroom teachers compared to female non-homeroom teachers, the aOR increased to 1.86 (0.99–3.61). As class hours increased, aOR tended to increase and was higher for females only but was not statistically significant. There was no statistically significant difference by school type and smoking after adjustment.

**Table 4:** Results of multiple logistic regression for frequent voice disorder (fVD): secondary school teachers

General and work-related factors	Total		Female	
	aOR^a^	95% CI	aOR^b^	95% CI
School type				
Middle	1.27	(0.63–2.59)	1.41	(0.61–3.42)
General high	0.99	(0.50–2.01)	1.03	(0.43–2.60)
Vocational high	1		1	
Age (yrs)				
< 30	1		1	
30–39	1.49	(0.54–4.82)	1.38	(0.50–4.61)
40–49	1.47	(0.52–4.89)	0.98	(0.31–3.44)
≥ 50	1.87	(0.68–6.14)	1.87	(0.64–6.40)
Sex				
Male	1		–	
Female	3.04	(1.42–7.26)^c^	–	
Smoking status				
Never smoker	1		–	
Ex-smoker	0.55	(0.18–1.59)	–	
Current smoker	0.96	(0.30–2.91)	–	
Sleep hours (hrs/day)				
< 6	1.42	(0.71–2.82)	1.15	(0.52–2.51)
6–7	1.18	(0.66–2.17)	0.96	(0.49–1.91)
≥ 7	1		1	
Class hours (hrs/wk)				
< 16	1		1	
16–19	1.33	(0.66–2.94)	1.65	(0.68–4.66)
≥ 20	1.90	(0.80–4.73)	2.37	(0.82–7.58)
Working hours (hrswk)				
40	1		1	
41–52	0.96	(0.49–1.98)	0.98	(0.46–2.19)
> 52	1.03	(0.38–2.73)	0.94	(0.28–3.01)
Homeroom teacher				
Yes	1.52	(0.90–2.62)	1.85	(0.98–3.60)
No	1		1	
Subject				
Physical education	1.03	(0.16–3.95)	–	
Music	0.60	(0.09–2.39)	–	
Others	1		–	
Sick leave as needed				
Yes	1		1	
No	2.40	(1.41–4.16)^c^	1.93	(1.03–3.73)^c^

*aOR* adjusted odds ratio, *95% CI* 95% confidence interval

^a^Adjusted for school type, age, sex, smoking, region, number of students, sleep hours, class hours, working hours, homeroom teacher, subject, and sick leave as needed

^b^Adjusted for school type, age, region, number of students, sleep hours, class hours, working hours, homeroom teacher, and sick leave as needed

^c^Statistically significant (*P* < 0.05)

## Discussion

This nationwide survey identified the effects of working conditions on VDs among randomly selected school teachers. In a nationwide on-line survey of primary and secondary teachers (*N* = 1879, 72.6% females) in New Zealand, 1 year prevalence of voice problem (having a vocal problem every couple of months or more often in 2010) was 24.7% [21]. A systematic review about voice disorders in teachers shows that several work-related factors were associated with VD, such as noise in classrooms, physical education instructor, weekly class hours, work pressure, and habitual use of a loud speaking voice [22].

Mechanically, voice production involves complex fluid-structure interaction within the glottis and its control by laryngeal muscle activation. The pathophysiology of hoarseness (impaired voice production) is characterized by muscle tone–related irregularity in the vibration of the vocal cords. The causes of hoarseness are diverse: functional dysphonia (30%), vocal cord nodule (10.7–31%), manifestation of internal disease, neurological diseases, psychogenic dysphonia, and organic dysphonia such as laryngitis (acute 42.1%, chronic 9.7%), benign tumors (10.7–31%), and malignancy (2.2–3%). Except for infections, malignancy and smoking, one of the major causes of these is phonotrauma, which is microvascular trauma with local edematous remodeling processes and accompanying inflammation as a result of misuse of the voice [23].

### Sick leave, class hours, and working hours

Several studies have noted an association between working conditions and VD. Teachers’ absenteeism due to VD is frequent, and the related costs are high [19]. However, presenteeism seems to be a bigger problem than absenteeism in Korea. This study showed that about half of survey teachers had difficulty in applying for sick leave as needed and those had higher risk than teachers who had no difficulty in applying for sick leave as needed. This might be a consequence of the nature of the teachers in feeling responsible for their students. However, Korea has a patriarchal organizational culture and lacks substitute teachers [1], which makes it difficult to apply for sick leave. In this situation, it is even more difficult to use sick leave due to VD which is non-serious disease and more common in teachers. Thus, it is considered that VDs become more frequent and chronic as teachers continue to use their voices without rest.

The results of this study suggest that class hours and working hours could be associated with VD, although not in a statistically significant manner. In a cross-sectional study of 2103 female teachers in Brazil, over 22.5 class hours per week was related to dysphonia; it showed a crude OR of 1.74 (1.21–2.49) compared to less than 22.5 class hours per week [24]. Consistent with this, our study showed that more than 20 class hours per week was associated with VD in secondary school teachers; it showed a aOR of 1.90 (0.80–4.73) compared to less than 16 class hours per week. However, VD of primary school teachers showed no significant difference according to class hours. A possible explanation for this might be that because most of primary school teachers have similar class hours, we were not able to identify a significant difference between them. Although not found in this study, not only the length of class but also the arrangement of class, which was included reducing consecutive class and giving sufficient time of rest, can affect VD because dose-response relationships were observed between frequency, duration of loud talk and VD [25]. In addition, a review conclude that negative changes in measuring vocal function appeared after 1–2 h of continuous voice use [26].

In a case-control study on teachers (case 40, control 40), the frequency of frequent vocal symptoms was significantly higher in the 40+ working hours group [27]. Consistent with that, our study found that the aOR of 52+ working hours was 2.70 (0.83–8.07) compared to that of 40 working hours among primary school teachers. The correlation was low between class hours and working hours (*r* = 0.0094), and it is considered that the possibility of over-adjustment is low in multiple logistic regression. There is little literature on the effects of long working hours on vocal health. The OR of the teachers’ voice complaints who self-reported high work pressure was 3.52(1.30–9.55) [28]. Some studies suggest that psychological stress is associated with vocal symptoms through the sympathetic nervous systems and elevated laryngeal muscle tension [29–31]. Teachers may use their voices a lot during long working hours, but it seems that long working hours themselves also act as stressors and affect vocal health. Under the Korean Labor Standards Act, teachers must work less than 40 h per week and up to 52 h per week if agreed upon by all parties. Nevertheless, 13.5% of total secondary school teachers and 21.1% of general high school teachers were working more than 52 h per week, especially those in charge of the third grade of general high school represents 31.6% of them. Long working hours are associated with various adverse health effects [32–36], and making teachers work over 52 h per week is a violation of the Korean Labor Standards Act. Therefore, measures are needed to control long working hours.

### Other working conditions

Homeroom teachers were reported to use their voices more often and to have a greater work burden because of providing student counseling and guidance in addition to classes [1]. This is thought to be one of the reasons that homeroom teachers in secondary school showed higher fVD. There was not shown this significant difference in primary school teachers, which is probably because 82.4% of them are homeroom teachers. Teachers who engage in vocally intense activities, such as loud talking and singing, were associated with a greater risk of developing a VD [37]. Consistent with literature, we found that fVDs were associated with music teachers in primary school. However, the results of music teachers in secondary schools and of physical education teachers did not reveal significant differences, presumably due to insufficient fVD numbers (*N* = 2). Previous studies on teaching careers and VDs are inconsistent [24, 38, 39]; our results showed no significant differences between teaching careers and fVD.

Although using a microphone was not included in multiple logistic regression in this study because the temporal relationship was unclear, the frequency of using a microphone was 16.1% (primary school teachers; 6.0%, secondary school teachers; 23.6%, respectively), and it seemed to be a risk factor of fVD; crude OR = 2.33 (0.95–5.20) among primary school teachers, 3.15 (1.94–5.09) among secondary school teachers, respectively. However, this is thought to involve reverse causality. A previous review noted that crowded classrooms and excessive noise are undoubtedly risk factors contributing to the development of dysphonia in teachers, and noisy classroom may be estimated at 58–90.5 dB [9]. A previous study reported an improvement of 13 dB in the voice intensity of teachers who use amplifiers [40]. Therefore, it seems necessary to use a microphone.

### Sex and sleep hours

Epidemiological evidence from previous studies is consistent with our results that the prevalence and risk of female VDs is higher than that of males. This result can be explained by structural gender differences in laryngeal anatomy. Females have shorter vocal folds (thus a higher speaking fundamental frequency) and lower concentration of hyaluronic acid, which is important for wound repair and shock absorption, in the superficial layer of the lamina propria [25, 41]. In the results of the secondary school teachers, the aORs of class hours and homeroom were higher in female teachers than for all teachers, but it was not statistically significant.

One Korean study using 2010–2012 data from the Korean National Health and Nutrition Examination Survey(KNHANES) revealed that 5 sleep hours or less was significantly associated with self-reported dysphonia and long-term dysphonia compared to 7 sleep hours: aOR = 1.45 (1.15–1.83), ≥ 3 weeks aOR = 1.57 (1.20–2.25), respectively [42]. Another study of teachers showed that there was a significant association between hoarseness and less than 6 sleep hours after adjustment for teaching career [43]. Our study also found that sleep hours were significantly associated with fVD. An association between fatigue and functional dysphonia was reported in a previous study [44]. Fatigue caused by sleep deprivation can lead to voice changes, followed by poor vocal performance, and VDs [45]. Further research is required because many factors can affect sleep hours.

### Limitations and strengths

There are several limitations to this study. First, our findings are limited by the use a cross- sectional design, so reverse causation could not be excluded. For example, a teacher with fVD might have reduced class or work. To minimize these effects, we asked questions about working conditions and voice symptoms which are limited to last one year. Second, the response rate was not high (48.3%), and we did not get information from non-participation. So there may be concern about selection bias. However, generally, if the response rate of the survey is more than 50%, it is evaluated as a positive level [46]. Considering that the response rate of mail survey is lowest among surveys [46], and the response rates for epidemiologic studies have been declining with even steeper in recent years [47, 48], the response rate of our study (48.3%) can be evaluated as positive level. Third, because the outcome was self-reported by participants, it may have been over-reported. Considering that, we allowed the participants to exclude respiratory infections when answering questionnaires and defined fVD as symptoms more than once a week. In two studies using video laryngoscopy for VD diagnosis, the point prevalence of teachers was high at 33 and 57% [49, 50]. Point prevalence of self-reported vocal symptoms by teachers was 9–37% in previous studies. Although our findings differed with regard to point prevalence, the proportion of the outcome was 11.61% (and 27.98% for more than twice a month), which is not high compared to previous studies. Self-reported symptoms are also meaningful as an outcome because voice change itself can affect a teachers’ work ability without a clinical diagnosis. Forth, there was insufficient consideration of environmental factors. Previous studies reported that working environment factors such as acoustics and noise condition, ventilation, lighting, temperature, and humidity are related to teachers’ VD [22]. In most of the previous studies on teachers’ VD and work-related factors, measurements were not performed because of difficulties. In this study, to take into account the working environment, we adjusted the average number of students per class (due to the acoustic and noise condition) and the region of school (urban and rural) when performing multiple logistic regression.

Despite these limitations, our study has strengths. First, this study is the first in Korea to investigate teachers’ working conditions and VD nationwide. In 2006, the Wonjin Institute of Occupational and Environmental Health (WIOE) and Korean Teachers and Education Workers Union (KTU) conducted a survey on working conditions and health for 2254 teachers, but it was limited to the Seoul Metropolitan City [51]. The Korea Ministry of Education has published a number of statistics annually, such as the *Statistical Yearbook of Education and International Statistics*. However, data on the teachers’ health status and specific working conditions (e.g., actual working hours, sick leave application status) are not included. Second, we divided schools into eight groups considering region and school type, and then randomly selected schools for each group. Next, we conducted a survey of all the teachers belonging to the randomly selected schools. Through this process, we tried to reduce the possibility of selection bias. The results identified in our data are similar to the results of all Korean teachers, as described in the *Statistical Yearbook of Education 2016* by Korea Ministry of Education, in which the average number of students per class was 22.4 for primary schools, 27.4 for middle schools, and 29.3 for high schools [20]. The average number of students per class in this study was 22.9 (SD 4.18) for primary schools, and 28.03(SD 4.69) for secondary schools. In the *Statistical Yearbook of Education 2016* by Korea Ministry of Education, the average class hours per week were 21.2 h for primary schools, 18.1 h for middle schools, and 16.8 h for high schools. The average class hours per week in this study data were 21.23 h (SD 3.90) for primary school, and 17.19 h (SD 2.70) for secondary school [20]. Third, we investigated the specific working conditions of the teachers and identified the associations with VD after stratifying and adjusting for related variables. Although many studies have examined teachers’ VDs, studies on the association between specific working conditions and VDs are rare, especially those including working and sleep hours. Moreover, much of the literature has yielded crude OR or aOR (e.g., age, sex, teaching career).

### Suggestions for reducing teacher’s voice disorders

Both structured and personal approaches are required to reduce teachers’ fVDs. Considering the structural approach first, it seems necessary to modify the length and arrangement of working and class time to prevent VDs, and to ensure appropriate break and sleep time through these modifications. For homeroom and music teachers, additional measures are needed. VDs were significantly lower in teachers who received voice training/education for more than 10 h [21]. A systematic, regular education program on appropriate vocal training and speech use is needed for education college students and teachers, along with a structured approach to the appropriate treatment of VDs. It is important for teachers to apply for sick leave when necessary and to create conditions and cultures that allow them to be treated and rested. Currently in Korea, voice-related disorders are not legally accepted as work-related diseases. Thus, policies that provide financial support and proper management are needed as work-related disease. At a personal level, a randomized clinical trial described that the control of voice use, the avoidance of behavior that could impair the vocal cords, the ingestion of water and changes in lifestyle were basic voice hygiene behaviors [52]. To control voice use, using a microphone would be beneficial. Finally, periodic health surveys and examinations are needed to find the cause of teachers’ VDs and to identify trends and other health effects.

## Conclusions

This study of teachers across Korea showed that teachers’ working conditions and sleeping hours were associated with fVD. Teachers’ health is not much addressed in school health and is largely left to the teachers themselves. Prevention through systematic regular education programs, early diagnosis, and appropriate treatment of VD is required. In order to do this, it is required to make a human resources structure and culture in the education system that can modify working conditions and provide sick leave more easily as needed, along with including VD as a legal work-related disease. This cross-sectional study has significant implications that regular follow-ups of teachers’ working conditions and health status are needed. This is necessary to identify changes in vocal and other health status and establish policies to improve teachers’ health.

## Abbreviations

AOR: Adjusted odds ratio
CGRI: Chamgyoyook Research Institute; class hours per week: the average of class hours per week during this semester; working hours per week: the average working hours per week during this semester
fVD: Frequent voice disorder
ILO: International Labour Organization
IRB: Institutional Review Board
KNHANES: Korean National Health and Nutrition Examination Survey
NHIS: National Health Insurance Service
OR: Odds ratio
SD: Standard deviation
U.S.: United States
VD: Voice disorder
